# Supplementary material for: Therapeutic Potential of Tumor Metabolic Reprogramming in Triple-Negative Breast Cancer
Source: Int J Mol Sci. 2023 Apr 8;24(8):6945. doi: 10.3390/ijms24086945 (PMC10138520; doi:10.3390/ijms24086945)
Supplement: Supplementary file 1 [file ijms-24-06945-s001.zip › ijms-2284426-supplementary.pdf]

Table S1. Ongoing clinical trials focusing on tumor metabolism administered to breast cancer patients, including those with triple-negative breast cancer. The 37 recruiting or soon-to-be-active interventional clinical studies involve chemotherapies, immunotherapies, hormone therapies, metabolic inhibitors, fasting-mimicking diets and vitamins.

NCT Number	Acronym	Title	Status	Conditions	Drugs	Phases	No. of patients
NCT03179904		FASN Inhibitor TVB-2640 and Trastuzumab in Combination With Paclitaxel or Endocrine Therapy for the Treatment of HER2 Positive Metastatic Breast Cancer	Recruiting	Advanced Breast Carcinoma, HER2 Positive Breast Carcinoma, Stage III-IV Breast Cancer	Anastrozole, Exemestane, FASN Inhibitor TVB-2640 Fulvestrant, Letrozole Paclitaxel, Trastuzumab	II	80
NCT04720664	OASIS	Oral SM-88 in Patients With Metastatic HR+/HER2- Breast Cancer (OASIS)	Active, not recruiting	Breast Cancer, Metastatic Breast Cancer, HR+ Breast Carcinoma	SM-88	II	50
NCT05736367		Investigating the Metabolic Pathways in Hormone Receptor Positive/HER2 Negative Breast Cancer	Not yet recruiting	Breast Cancer	Glucose (U-13C-glucose)	I	16
NCT05763992	BREAKFAST-2	Targeting Triple Negative Breast Cancer Metabolism With a Combination of Chemoimmunotherapy and a FASTing-like Approach in the Preoperative Setting: the BREAKFAST 2 Trial	Not yet recruiting	Breast Cancer, Triple Negative Breast Cancer	Control diet, Fasting-Like Approach	II	145
NCT03971019	SBSBC	Survival Benefits of Statins in Breast Cancer Patients	Recruiting	Breast Cancer	Statins	III	314
NCT04248998	BREAKFAST	Calorie Restriction With or Without Metformin in Triple Negative Breast Cancer	Active, not recruiting	Triple Negative Breast Cancer	Fasting-mimicking diet, Metformin + Preoperative chemotherapy	II	30

NCT05010356		Insulin Sensitivity After Breast Cancer	Recruiting	Breast Cancer	Insulin	NA	24
NCT05570253		A Study of SDX-7320 in Combination With Eribulin for People With Breast Cancer	Recruiting	Breast Cancer, Metastatic Triple Negative Breast Cancer	Eribulin, SDX-7320	II	55
NCT05067530[1]	CAREGIVER	Cyclin dependent Kinase in triple negative breast cancer - a "Window of Opportunity" Study	Not yet recruiting	Triple Negative Breast Neoplasms	Palbociclib, Paclitaxel, Carboplatin	II	126
NCT02317783		Amyloid Plaque Deposition in Chemotherapy-Induced Cognitive Impairment	Recruiting	Breast Cancer	[18F]Flutemetamol, [18F]fluoro-2-deoxy-D-glucose (FDG)	II	15
NCT05455619	Amelia-1	Evexomostat Plus Alpelisib and Fulvestrant in Postmenopausal Women at Risk for Hyperglycemia With Breast Cancer	Recruiting	HR+/HER2-negative Breast Cancer, Metastatic Breast Cancer	Evexomostat	I/II	52
NCT03161353	PHERGain	Chemotherapy-free Trastuzumab and Pertuzumab in HER2-positive Breast Cancer: FDG-PET Response-adapted Strategy.	Active, not recruiting	Breast Cancer	Perjeta, Herceptin, Docetaxel, Carboplatin, Letrozole, Tamoxifen	II	377
NCT02546232		Improved Breast Cancer Therapy (I-BCT-1) in the Neoadjuvant and Metastatic Setting	Active, not recruiting	Breast Cancer	Carboplatin, Paclitaxel	II	196
NCT05716516	ESTHER	STUDY02001740;22SCH740: Estradiol For ER+ Advanced Breast Cancer (ESTHER)	Not yet recruiting	Metastatic Breast Cancer	Estradiol	II	36

NCT05501158		CYP2D6 Genotypes and Breast Cancer Clinical Outcomes in the Indonesian Population	Active, not recruiting	Breast Cancer	Tamoxifen	NA	150
NCT03121989	LABC	Study to Evaluate the Feasibility of 13-C Pyruvate Imaging in Breast Cancer Patients Receiving Neoadjuvant Chemotherapy	Recruiting	Breast Cancer	Hyperpolarized Pyruvate (13C), NAC	I	13
NCT01419730		Vitamin D and Physical Activity on Bone Health	Active, not recruiting	Breast Cancer	Vitamin D3	II	191
NCT04483505	ROGABREAST	Rogaratinib, Palbociclib and Fulvestrant in Patients With Breast Cancer.	Active, not recruiting	Metastatic Breast Cancer, HR+ Malignant Neoplasm of Breast	Rogaratinib, Palbociclib, Fulvestrant	I	19
NCT01565200	ZEPHIR	HER2 Imaging Study to Identify HER2 Positive Metastatic Breast Cancer Patient Unlikely to Benefit From T-DM1	Active, not recruiting	HER-2 positive Breast Cancer	T-DM1, Trastuzumab	II	90
NCT03934905		Protective Effects of the Nutritional Supplement Sulforaphane on Doxorubicin-Associated Cardiac Dysfunction	Recruiting	Breast Cancer	Sulforaphane	I/II	70
NCT03324932		Efficacy of Denosumab on Normal BMD in Women Receiving Adjuvant Aromatase Inhibitors for Early Breast Cancer	Recruiting	Breast Cancer	Denosumab	III	160

NCT01273168		Endoxifen in Adults With Hormone Receptor Positive Solid Tumors	Active, not recruiting	HR+ Breast Cancer	Endoxifen	I	40
NCT05759949		First-in-Human Study of RLY-5836 in Advanced Breast Cancer and Other Solid Tumors	Not yet recruiting	PIK3CA Mutation, HER2-negative Breast Cancer, Metastatic Breast Cancer, Advanced Breast Cancer, HR+ Breast Cancer	RLY-5836[2], Fulvestrant, Palbociclib, Ribociclib, Abemaciclib	I	220
NCT05768139		First-in-Human Study of STX-478 as Monotherapy and in Combination With Other Antineoplastic Agents in Participants With Advanced Solid Tumor	Recruiting	Breast Cancer|Gynecologic Cancer|HNSCC|Solid Tumors	STX-478, Fulvestrant	I/II	160
NCT04862663	CAPItello-292	Capivasertib + Palbociclib + Fulvestrant for HR+/HER2- Advanced Breast Cancer (CAPItello-292).	Recruiting	Locally Advanced (Inoperable) or Metastatic Breast Cancer	Capivasertib[3], Fulvestrant, Palbociclib	III	700
NCT02592083	PREDIX LumA	Neoadjuvant Response-guided Treatment of Slowly Proliferating Hormone Receptor Positive Tumors	Active, not recruiting	Early-Stage Breast Carcinoma, HR+ Tumor	Tamoxifen or Aromatase Inhibitor or Aromatase Inhibitor + Goserelin, Palbociclib	II	10
NCT01992952	FAKTION	Fulvestrant +/- Akt Inhibition in Advanced Aromatase Inhibitor Resistant Breast Cancer	Active, not recruiting	Estrogen Receptor Positive Breast Cancer	AZD5363, Fulvestrant	I/II	149

NCT03284957	AMEERA-1	Phase 1 / 2 Study of Amcenestrant (SAR439859) Single Agent and in Combination With Other Anti-cancer Therapies in Postmenopausal Women With Estrogen Receptor Positive Advanced Breast Cancer	Active, not recruiting	Estrogen Receptor Positive Breast Cancer	Amcenestrant, Palbociclib, Alpelisib, Everolimus, Abemaciclib	I/II	136
NCT05483491		KK-LC-1 TCR-T Cell Therapy for Gastric, Breast, Cervical, and Lung Cancer	Recruiting	Gastric Cancer, Breast Cancer, Cervical Cancer, Lung Cancer	KK-LC-1 TCR-T cells, Aldesleukin	I	42
NCT01357772	TAM-01	Trial of Low Dose Tamoxifen in Women With Breast Intraepithelial Neoplasia - Long Term Follow-up	Active, not recruiting	Breast Neoplasms	Tamoxifen	III	500
NCT05753657		A Pilot Study of Monitoring Insulin Levels and Treating Hyperinsulinemia and Hyperglycemia With Pioglitazone in Patients Treated With Alpelisib for Metastatic Breast Cancer.	Recruiting	Metastatic Breast Cancer|Hyperinsulinism|Hyperglycemia Drug Induced	Pioglitazone	I	30
NCT03343054		A Phase 1 Study Of Talazoparib, PARP Inhibitor, In Japanese Patients With Advanced Solid Tumors	Active, not recruiting	Neoplasms, Breast Neoplasms	Talazoparib	I	28

NCT04247126		A Study of SY 5609, a Selective CDK7 Inhibitor, in Advanced Solid Tumors	Active, not recruiting	Advanced Solid Tumor, Breast Cancer, Small-cell Lung Cancer, Pancreatic Cancer	SY-5609, Fulvestrant, Gemcitabine, Nab-paclitaxel	I	160
NCT04004910		A Study Comparing Immunopheresis Alone or In Combination With Chemotherapy Versus Chemotherapy Alone in Treatment of Advanced Breast Cancer Patients	Recruiting	Advanced Breast Cancer	Plasma soluble TNF receptor pulldown + chemotherapy	I/II	170
NCT04395339	GLORY	GM1 Prophylaxis for WBRT Related Cognitive Dysfunction	Recruiting	Brain Metastases, Breast Cancer	Monosialotetrahexosyl ganglioside (GM1)	III	204
NCT02632448		A Study of LY2880070 in Participants With Advanced or Metastatic Cancer	Recruiting	Solid Tumors	LY2880070[4], Gemcitabine	I/II	229
NCT02000089	CAPS5	The Cancer of the Pancreas Screening-5 CAPS5)Study	Recruiting	Breast Cancer and Pancreas Cancer	Secretin	III	7000

[1] Primary endpoint, difference in early (i.e., after three weeks of therapy, 1 cycle) metabolic response. [2] RLY-5836 is a PI3Kα Inhibitor. [3] Capivasertib is an oral serine/threonine kinase inhibitor, as an inhibitor of protein kinase B. Capivasertib affects the PI3K/Akt/mTOR signaling pathway. [4] LY2880070 is an oral, selective competitive inhibitor of checkpoint kinase 1.
